# Alkali-Activated Slag Paste with Different Mixing Water: A Comparison Study of Early-Age Paste Using Electrical Resistivity

**DOI:** 10.3390/ma13112447

**Published:** 2020-05-27

**Authors:** Yubin Jun, Young Hwan Bae, Tae Yong Shin, Jae Hong Kim, Hong Jae Yim

**Affiliations:** 1 Department of Civil and Environmental Engineering, Korea Advanced Institute of Science and Technology, Daejeon 34141, Korea; ssjun97@gmail.com (Y.J.); tyshin@kaist.ac.kr (T.Y.S.); jae.kim@kaist.ac.kr (J.H.K.); 2 Department of Civil Engineering, Pusan National University, Busan 46241, Korea; onlybyh@gmail.com

**Keywords:** Alkali-activated slag, seawater, electrical resistivity, XRD, early strength

## Abstract

This paper reports the electrical resistivity measurements on KOH-activated ground-granulated blast-furnace slag, which was mixed with deionized water or natural seawater at three different activator-to-binder ratios (0.4, 0.45, and 0.5). Compressive strength and X-ray diffraction analyses were performed on the samples after the measurement. The type of mixing water did not affect the setting time of samples, whereas the setting time was delayed with an increase in activator-to-binder (*a/b*) ratio. Regardless of the mixing water type, the increasing ratio of electrical resistivity between *a/b* 0.45 and 0.5 was larger than that between *a/b* 0.4 and 0.45. For the same *a/b* ratio, the pastes mixed with seawater produced higher electrical resistivity and early strength than those with deionized water. The increase in the electrical resistivity in seawater-mixed pastes could be attributed to the formation of Cl-bearing phases such as Cl-hydrocalumite, AlOCl, and aluminum chloride hydrate. It is believed that the reaction products in seawater-mixed samples were helpful in preventing water percolation, and thus, the electrical resistivity increased compared with the deionized water-mixed sample.

## 1. Introduction

As CO_2_ is emitted during cement manufacturing process, alkali-activated slag has been considered as an alternative structural binder. The alkali-activated slag is generally synthesized by mixing blast furnace slag with alkaline solutions. Blast furnace slag is a steel industrial by-product that is recycled in cement and concrete in many ways [1,2,3]. It is reported that the alkali-activated slag shows better mechanical properties compared with ordinary Portland cement [4,5,6]. Regardless of the binder types (cement-based or alkali-activated binder), their hardened states are governed by the properties of their fresh state, as their hydration induces microstructural evolution with water. This phenomenon is accompanied by generating hydration products with decreasing porosity [7]. Various methods were adopted to measure the degree of hydration by microstructural development, which include isothermal calorimetry, X-ray diffraction, mercury intrusion porosimetry, and infrared spectroscopy [8,9]. In accordance with microstructural evolution, the critical time to indicate the onset of the transition from suspension to solid material and strength arise is defined as setting time. Several conditions, such as used material types, mixed proportions, and environmental conditions for curing, affect the setting time. Further, these parameters determine the hardening of materials.

To evaluate the setting time and hardening of cement-based materials, various nondestructive techniques have been proposed using ultrasound inspections and electrical estimation, including the Vicat needle test [10,11,12]. Among them, electrical estimation is one of the simpler and promising methods, owing to its superior sensitivity in suspension and quick measurements with a cost-effective testing setup. The electrical estimation method measures the electrical conductivity and resistivity. The change in electrical properties is determined by the current flow caused by ion transport through the water-filled porosity in suspension. This characteristic has been used to investigate the effect of admixtures in cement-based mixtures based on the measurements of electrical resistivity [13,14], and a saturating condition in cement paste and mortar was considered to measure an electrical resistivity [15,16]. It has been applied to hydration monitoring [10,17,18,19,20]. To prevent the erroneous effect of electrode contact resistance in cement-based materials, the four-electrode method was used and electrical resistivity was monitored [10]. Electrical estimation can nondestructively evaluate the generating hydration products and monitor a degree of hydration in early-age suspension. This study intends to address this electrical estimation method to monitor microstructural evolution of the alkali-activated slag. It is reported that physical and mechanical properties (workability, durability, and strength) of the alkali-activated slag are affected by various factors, such as types of activator, slag, and fly ash, as well as activator concentration, activator-to-binder ratio, and curing condition [5,21,22,23]. This paper reports the results of electrical resistivity measurements on alkali-activated slag, which was mixed with two different types of mixing water and different activator-to-binder ratios. Further, this paper reports on the relation between the hardening and mineralogical properties during the early stages of alkali activation. The electrical resistivity, compressive strength, and X-ray diffraction of alkali-activated slag pastes were analyzed. 

## 2. Experimental Details

### 2.1. Materials

**Figure 1:**
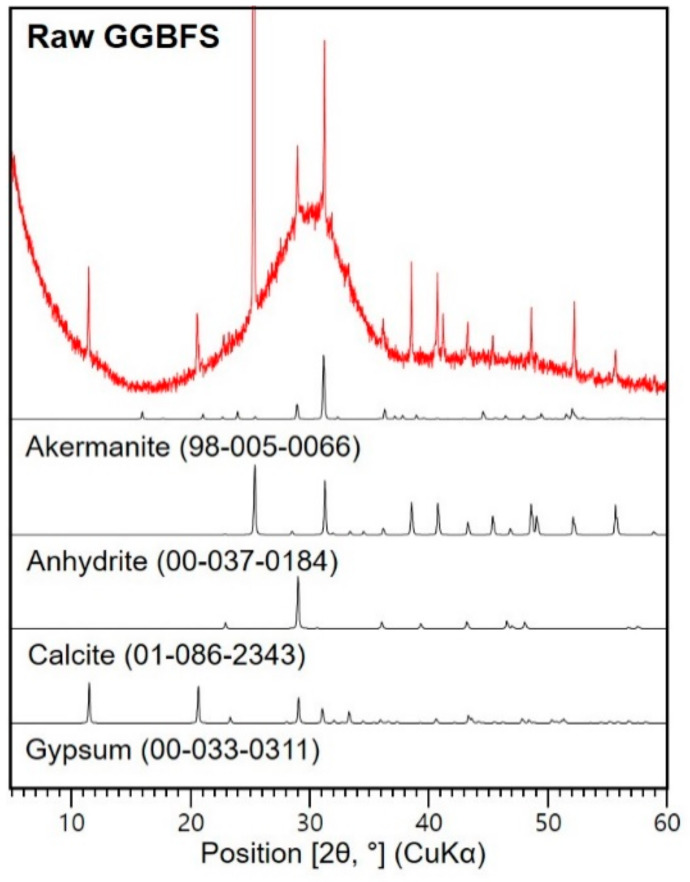
X-ray diffraction (XRD) patterns of raw ground-granulated blast-furnace slag (GGBFS).

A ground-granulated blast-furnace slag (GGBFS) was obtained. The chemical composition of the raw GGBFS was analyzed using X-ray fluorescence and presented in Table 1. Figure 1 shows the X-ray diffraction (XRD) patterns of the material with the reference peaks of the identified phases. The GGBFS had an amorphous phase with certain crystalline phases, such as akermanite, anhydrite, calcite, and gypsum. Anhydrite and gypsum were probably added during the milling process to meet Korean Standard (KS) F 2563 [24], in which (CaO + MgO + Al_2_O_3_)/(SiO_2_) is required to be greater than 1.60, for the chemical composition of commercial GGBFS powder. The added sulfate sources increase the SO_3_ content in the chemical composition of GGBFS. Potassium hydroxide (KOH; pellet, ≥85% purity) was used as the alkaline activator. Seawater was collected from Songjeong beach (35°10′47.5″ north, 129°12′17.9″ east) in Busan, South Korea. The chemical composition of the seawater is tabulated in Table 2. The cations were measured using inductively coupled plasma atomic emission spectroscopy and anions were quantified using ion chromatography.

### 2.2. Sample Preparation

GGBFS was mixed with a KOH solution. The KOH solution was synthesized by dissolving KOH pellets in seawater or deionized water to 4 mol/L and cooling to room temperature. The molarity of alkali activators plays a role in the alkali activation, and the higher activator concentration generally results in higher strength [5,25]. In this study, 4 M concentration instead of stronger concentration was chosen considering a safety problem and economical experimental condition. The weight ratios of the activator (4 M KOH in seawater or deionized water) to the binder (GGBFS) were set to 0.4, 0.45, and 0.5. Mixture proportion of the pastes are summarized in Table 3. The pastes were synthesized in a laboratory according to ASTM C305 [26].

### 2.3. Test Methods

#### 2.3.1. Electrical Resistivity Measurement

**Figure 2:**
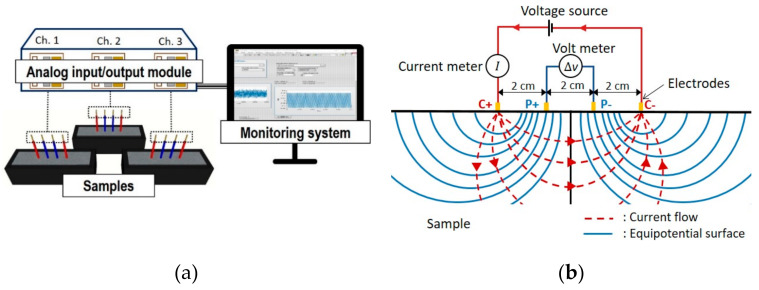
(**a**) Schematic experimental setup of electrical resistivity measurement; (**b**) schematic of the four-electrode method [10].

Wenner’s four-electrode method was used to monitor the electrical resistivity of alkali-activated GGBFS (AAS) paste, for 24 h, mixed with deionized water or seawater. This method was verified by previous experiments that measured the electrical characteristics of various types of cement-based materials without an electrode contact error [10]. The schematic experimental setup, four-electrode setup for electrical resistivity measurement, and four-electrode method are illustrated in Figure 2. 

The four electrodes used in this setup is divided into two parts: two electrodes on the inside for measuring current and two electrodes on the outside for measuring potential. The spacing of the electrodes was optimized at 20 mm [10]. Copper electrodes with a diameter of 1.78 mm were inserted at 20 mm from the surface to the middle of the sample. Approximately 10 mm at the end of each electrode were peeled off and contacted with sample. The AAS pastes were cast in molds with dimensions of 40 × 40 × 160 mm after mixing. The cuboid-shaped mold was fabricated with a non-conducting material (polyethylene). To prevent the polarization of water molecules and generate an alternating current, a sinusoidal potential at the outer current electrodes was created by a waveform generator (National Instrument 9263, National Instrument, Austin, TX, USA,). Here, its peak amplitude voltage and frequency were ±10 V and 500 kHz, respectively. To avoid an electrical charge during the alternating current generation, the duration of the electric potential was limited to 10 ms. Simultaneously, the potential difference (*V*) was measured using a voltage meter (National Instrument 9222, National Instrument, Austin, TX, USA) at the inner electrodes and the current (*I*) was measured at the outer electrodes using an alternating-current module (National Instrument, 9227). The electrical resistivity (*ρ = 2παR*) of the AAS pastes was then evaluated based on the measured electrical resistance by Ohm’s law (*R* = *V*/*I*). Here, α is 20 mm, which is the spacing between two electrodes. For each paste mix, three samples were synthesized for the sake of repeatability of measurement, and each measurement was conducted at an interval of 10 min during a 24 h monitoring. Relative humidity and temperature during the experiment were maintained at 50% and 25 °C, respectively.

#### 2.3.2. Compressive Strength Test

After mixing, the fresh pastes were cast in cubic molds with dimensions of 25 × 25 × 25 mm for compressive strength (Compression Testing Machine, PWS-400A, Woo Jin Co., Gyeonggi-do, Korea) testing. They were placed in the laboratory conducting the measurement of electrical resistivity. The samples were cured under 25 °C with 50% relative humidity. The compressive strength of the paste samples was measured in accordance with ASTM C109 [27] at 24 h after the completion of the electrical resistivity test. The loading rate was 1.0 kN/s. Each strength result shows an average value obtained from five identical samples.

#### 2.3.3. XRD

Fractured specimens after the compressive strength test were finely powdered and subjected to a solvent-exchange method using isopropanol to prevent further reactions [28]. After vacuum drying, the specimens were examined using XRD. The XRD analysis was performed on a high-resolution X-ray diffractometer (Bruker D8 DISCOVER, Billerica, MA, USA) with Cu-Kα radiation (λ = 1.5406 Å), and the XRD patterns were collected from 5° to 60° (2θ) at a scan rate of 2°/min. The XRD patterns were analyzed using X’pert HighScore Plus program [28] with the International Centre for Diffraction Data (ICDD)-Powder Diffraction File (PDF) [29] and the Inorganic Crystal Structure Database (ICSD) [30,31]. 

## 3. Results and Discussions

### 3.1. Electrical Resistivity

**Figure 3:**
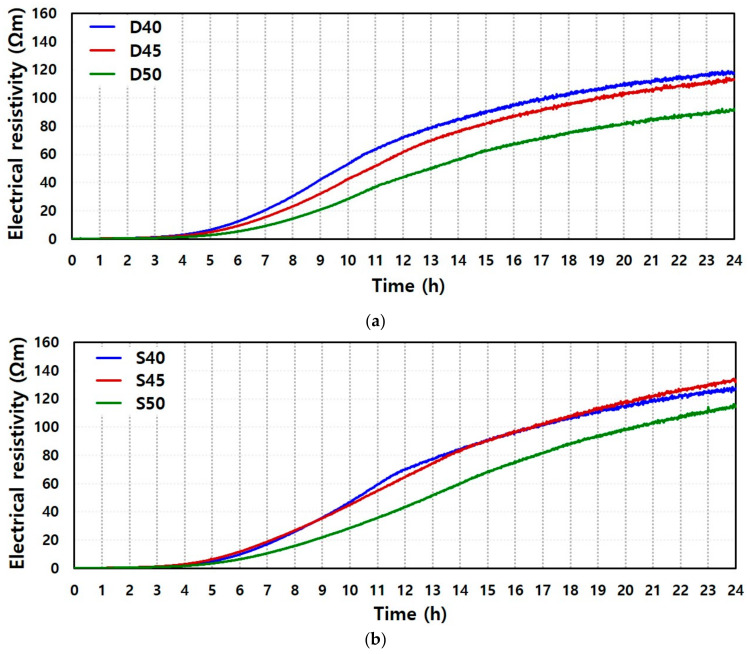
Comparison of measured electrical resistivity of (**a**) deionized water-mixed and (**b**) seawater-mixed AAS pastes with different *a/b* ratios; 40, 45, and 50 represent *a/b* ratios of 0.4, 0.45, and 0.5, respectively.

Figure 3 shows the 24 h measured electrical resistivity of AAS pastes that were mixed with deionized water (D40, D45, and D50; Figure 3a) and seawater (S40, S45, and S50; Figure 3b). 

The results represent the difference in electrical resistivity and are compared with different *a/b* ratios: 0.4, 0.45, and 0.5. The trend of electrical resistivity as a function of time follows the results of cement-based materials in a previous study [10]. It was reported that the measured electrical resistivity provides three parameters to characterize the microstructural evolution during hydration. These are identified as the initial resistivity, rising time to indicate the onset of an increase in electrical resistivity, and increasing ratio of electrical resistivity after the rising time. Current flow and measured electrical resistivity are determined using the water network through GGBFS particles. Therefore, the value of the initial electrical resistivity is established using the spacing of GGBFS particles with percolated water, which can reflect the initial microstructure of suspension as the *a/b* ratio. Here, the results of the initial resistivity of GGBFS-based alkali-activated pastes with seawater or deionized water is determined at an average time of 30 min, and its value is approximately 0.19 Ωm and 0.20 Ωm, respectively. Hence, the state of the initial microstructures of all the AAS pastes is similar and they exhibit sufficient electrical conductivity. For hours after mixing, microstructural change is not sufficiently large to affect the electrical resistivity. This is ascertained by the constant initial value of electrical resistivity observed for a few hours of the inactive period. However, the electrical resistivity reaches a critical point after 3 h and gradually increases. Here, a critical point is the rising time as an indicator of the setting time. This is because the coagulated GGBFS particles lead to a closing of water network and solid percolation. Further, the increasing ratio of electrical resistivity after the rising time describes the hardening phase of pastes owing to a solid network evolution caused by alkali activation.

In this study, the rising time is determined by the measured electrical resistivity that is 5 times higher than the initial value, and the increasing ratio of resistivity (Ωm/h) is the average slope of the curve after the rising time of 24 h. The calculated parameters are reported in Table 4. 

Comparing all the AAS pastes, it was observed that the rising time of the pastes with seawater or deionized water was delayed with higher *a/b* ratios (0.5) than with the lower *a/b* ratios (0.4 and 0.45). While the change in rising time was not high, the increased activator content tended to induce a delay in setting time. The pattern of delayed setting time was similar to the increasing resistivity after the rising time. Different types of mixing water and their mixing ratios influence the degree of alkali activation. This phenomenon demonstrates that a lower activator content leads to an increase of reaction products with capillary pores depercolation and cutting of conductive wires in a sample. Here, the state of sample was no longer the suspension. This supports the notion that the higher activator content improves the dispersion of GGBFS particles but does not influence the initial resistivity, and different activator content can induce a change in rising time and its increasing ratio. The changed ratio of both parameters between *a/b* 0.45 and 0.5 was larger than that between *a/b* 0.4 and 0.45. In particular, the trends of changed electrical resistivity between S40 and S45 was almost similar, it was hard to find the effect of *a/b* ratio on setting and hardening of GGBFS. This is because the optimized *a/b* ratio for hydration was between 0.4 and 0.45 for early age activation of GGBFS within 24 h, regardless of the mixing water type, and more water content remained as free water in the mixture. This influences the electrical resistivity and its increasing ratio, and leads to the difference in setting time and hardening process. 

**Figure 4:**
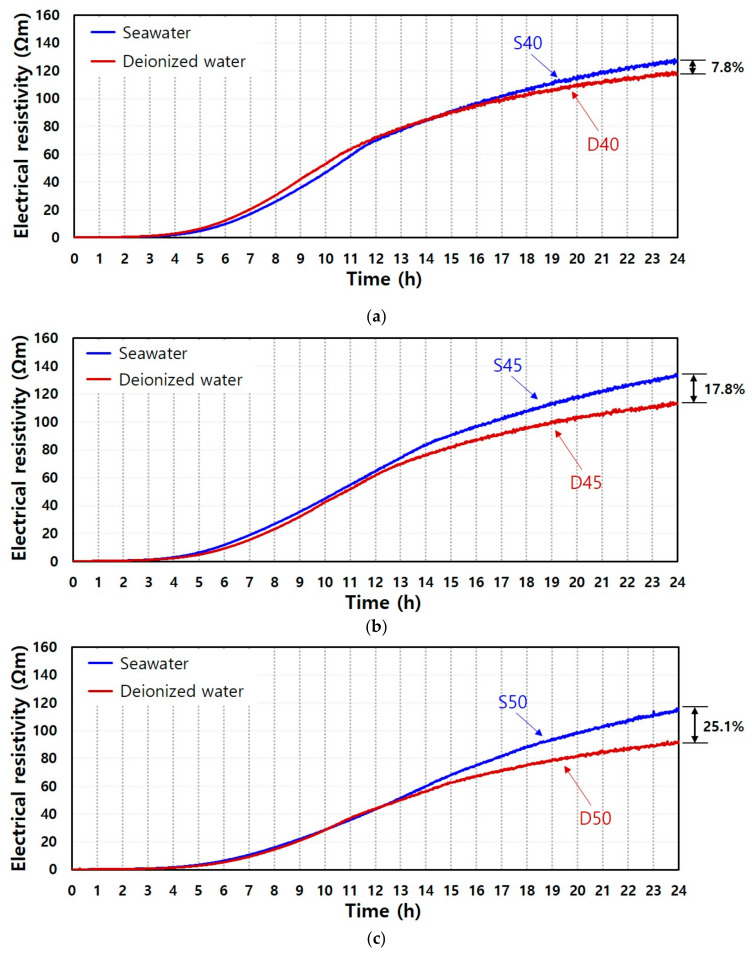
Comparison of measured electrical resistivity of seawater-mixed and deionized water-mixed AAS pastes with different *a/b* ratios: (**a**) 0.4, (**b**) 0.45, and (**c**) 0.5.

The results of three groups (*a/b* ratio of 0.4, 0.45, and 0.5) in Figure 4 demonstrate the effects of the types of mixing water. Figure 3 shows that the lower *a/b* leads to a higher electrical resistivity and faster hardening. It was observed that the rising time was similar in both pastes with the same *a/b* ratio, and this trend was represented especially in the increasing ratio of electrical resistivity until approximately 12 h after rising time. Hence, the type of mixing water does not significantly affect the setting time by the alkali activation of GGBFS, but the increasing ratio of electrical resistivity and values of electrical resistivity differed as a function of time until 24 h with different mixing water under the same *a/b* ratio. This supports the notion that an activation degree after setting time is dependent on the mixing water type, and seawater can better promote microstructural evolution of GGBFS than deionized water. Additionally, the difference in the degree of hardening was remarkable with higher *a/b* ratio. Here, the difference of electrical resistivity at 24 h under 0.4, 0.45, and 0.5 of *a/b* was 7.8%, 17.8, and 25.1%, respectively. The mixing water type rather than the dissolution and solid volume fraction of GGBFS particles can control the hardening of the AAS pastes.

### 3.2. Compressive Strength

**Figure 5:**
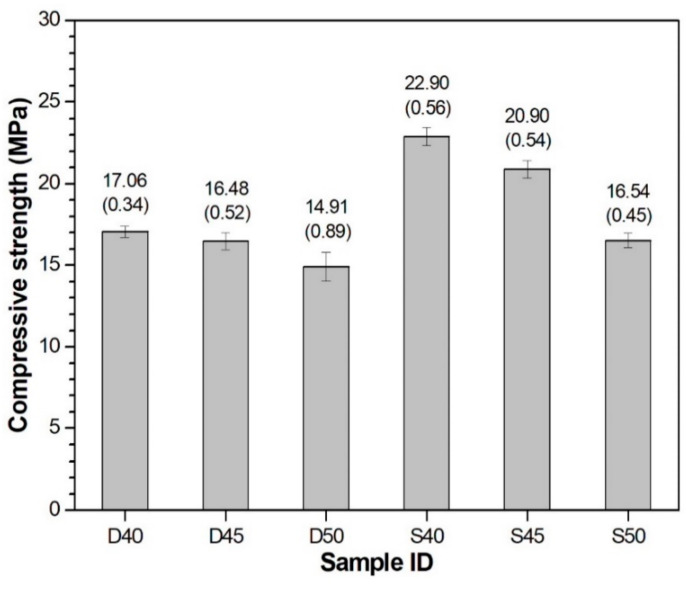
Compressive strengths at 24 h. The number above each bar indicates compressive strength testing result (standard deviation) in MPa.

The compressive strength results at 24 h of GGBFS-based alkali-activated pastes with seawater or deionized water are presented in Figure 5. 

The results show a decrease in compressive strength with increasing *a/b* ratio, irrespective of the type of mixing water. This result agrees with the previous observation on alkali-activated binder [25,32]. For each *a/b* ratio, the seawater-mixed AAS sample showed higher strength than the deionized water-mixed sample. In this study, the strength testing results are consistent with the electrical resistivity results. The samples arranged in the order of compressive strength from the lowest to highest were: D50, D45, S50, D40, and S45. Their electrical resistivity was 4.40, 5.43, 5.51, 5.67, and 6.30 Ωm/h, respectively, indicating that the sample exhibiting low electrical resistivity showed low compressive strength. Here, the S40 sample (22.9 MPa) showed higher strength than S45 sample (20.9 MPa), although the increasing ratio of resistivity for S40 (6.24 Ωm/h) was lower than that of S45 (6.30 Ωm/h). It might be because certain reaction products formed in S45 helped in the capillary pores depercolation. However, they do not contribute to the development of strength.

### 3.3. XRD Analysis

**Figure 6:**
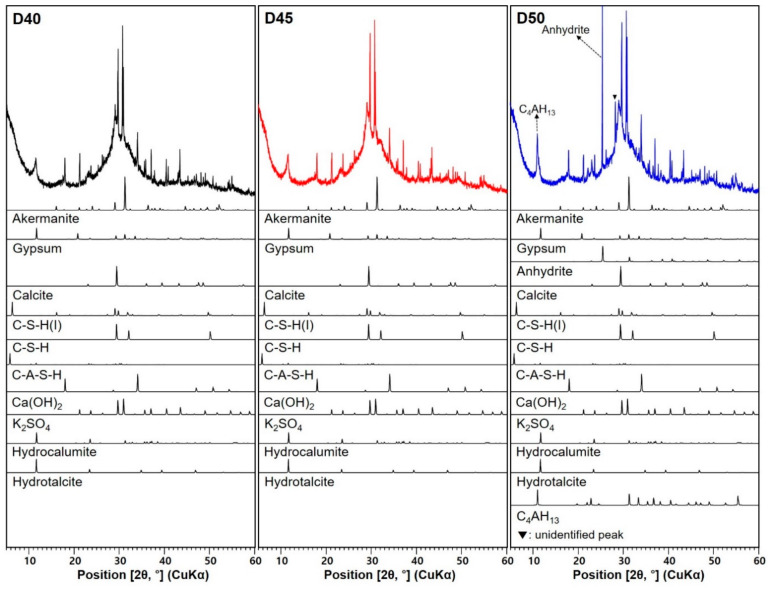
XRD patterns of deionized water-mixed AAS pastes with different *a/b* ratios.

**Figure 7:**
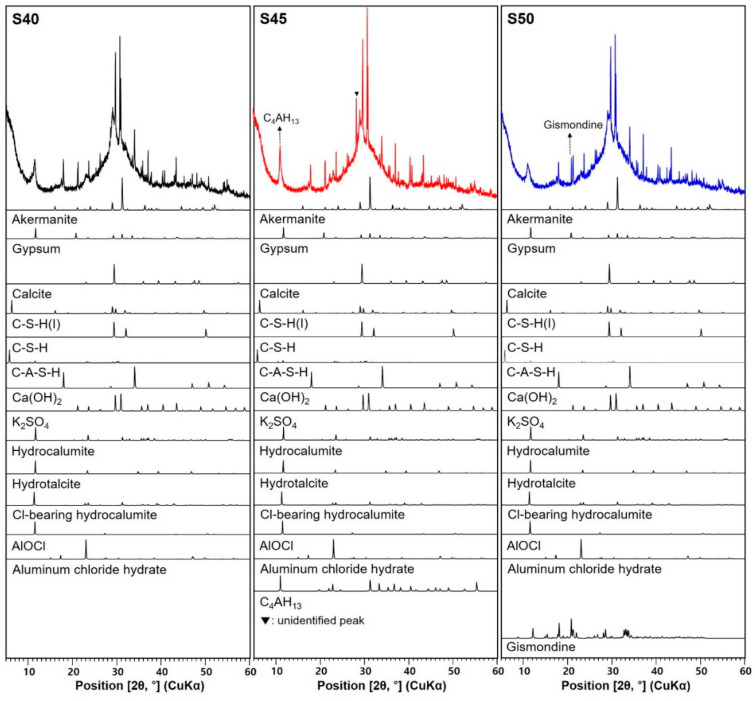
XRD patterns of seawater-mixed AAS pastes with different *a/b* ratios.

Figure 6 shows the XRD patterns of deionized water-mixed AAS pastes at 24 h after the electrical resistivity test. The phase changes observed in Figure 6 are listed in Table 5. For seawater-mixed AAS paste samples, XRD results and phase changes are presented in Figure 7 and Table 6, respectively. Studies [33,34,35,36] have reported that the main reaction products in alkali-activated slag are C–S–H(I), C–A–S–H(I), hydrogarnet, C_4_AH_13_, and hydrotalcite. In AAS paste samples with deionized water (D40, D45, and D50), various reaction products are identified, such as C–S–H(I), C–S–H, C–A–S–H, Ca(OH)_2_, K_2_SO_4_, hydrocalumite (3CaO∙Al_2_O_3_∙CaCO_3_∙11H_2_O), and hydrotalcite, which are also detected in seawater-mixed samples (S40, S45, and S50). In addition, akermanite, gypsum, anhydrite, and calcite contained were identified the raw GGBFS.

When the slag mainly consists of amorphous phase, it does not produce calcium hydroxide (Ca(OH)_2_) as a reaction product [37]. It is reported that Ca(OH)_2_ can be formed in CaO or Ca(OH)_2_-activated slag. XRD patterns of the original GGBFS (Figure 1) showed relatively strong gypsum and anhydrite peaks in comparison with existing literature [23,37,38]. Ca ions from gypsum and anhydrite can be consumed, producing a Ca-containing phase [39]. Considering that the reflection intensities of gypsum in AAS pastes were significantly decreased and the anhydrite was decreased or absent, it is believed that the formation of Ca(OH)_2_ in this study may be produced from the gypsum and anhydrite. It was expected that K_2_SO_4_ would be produced by the activator (KOH) and SO_4_^2−^ contained in seawater. However, considering that regardless of whether or not seawater was used, K_2_SO_4_ was detected in all samples. The sulfate from the gypsum and anhydrite contained in the raw GGBFS may have reacted with K^+^ in KOH to form K_2_SO_4_. This demonstrates that the reaction products of AAS can depend on certain crystalline phases caused by the GGBFS and type of activator. For each AAS sample with seawater or deionized water, the reflection intensities of K_2_SO_4_ increases as the *a/b* ratio increases, which is due to the increase in the amount of KOH solution per amount of binder.

D45 sample exhibited less C–S–H(I) and C–A–S–H than D40 (Table 5). It suggests that they would have affected the value of electrical resistivity between the two samples. D50 exhibited the newly formed phases (C_4_AH_13_ and unidentified peak). However, undissolved anhydrite remained in D50, but was absent in D40 and D45 (Figure 6). This suggests that the degree of D50 hydration was lower than that in the D45 and D50 samples. This may allow water percolation, and thus, cause the low electrical resistivity.

When the seawater was used in AAS, Cl-bearing hydrocalumite (3CaO∙Al_2_O_3_∙CaCl_2_∙10H_2_O), AlOCl (aluminium oxide chloride), aluminum chloride hydrate, and gismondine (CaAl_2_Si_2_O_8_∙4H_2_O) were formed, unlike in the deionized water-mixed AAS. Here, it can be observed that Cl-hydrocalumite, AlOCl, and aluminum chloride hydrate are reaction products pertaining to the chloride ions in seawater. Hydrocalumite belongs to a group of layered double hydroxides (LDHs), which exhibits an anion-exchange capacity [38,40]. The hydrocalumite formed in the seawater-mixed AAS paste is a Cl-exchanged phase with the strongest peak of 11.362° (2θ). It is reported that Cl in seawater can be present in the form of OCl^–^ under the alkaline environment [41,42]. It is expected that the presence of AlOCl in the seawater-mixed AAS may be due to the reaction between Al from GGBFS and OCl^–^ in the alkaline solution with seawater. Zeolites are generally observed in alkali-activation of fly ash [43]. Gismondine, which is a zeolitic aluminosilicate, was observed in alkali-activation of GGBFS and GGBFS/metakaoline blends based on sodium silicate solution and sodium hydroxide [44]. The observation of gismondine in S50 may indicate that the seawater-mixed AAS may contain a zeolitic phase. 

S45 sample showed relatively less reaction products (the reduction of C–S–H(I), C–S–H, Ca(OH)_2_, hydrocalumite, hydrotalcite etc.) than S40 (Table 6). However, the value of electrical resistivity of S45 was higher than that of S40, while the compressive strength was lower. This may imply that a specific reaction product is responsible for the capillary pores depercolation. However, it does not lead to the strength development. Here, the reaction products would be C_4_AH_13_ and the unidentified crystalline phase (Figure 7), as they were present in S45 but absent in S40. Although gismondine was formed only in S50, its XRD patterns showed relatively low peak intensities for the commonly identified reaction products in seawater-mixed samples. This would result in a low electrical resistivity in S50.

For each *a/b* ratio (0.40, 0.45, and 0.50), seawater-mixed AAS paste samples hardened faster than the deionized water-mixed AAS samples. This could be attributed to the crystalline phases formed using seawater. S40 exhibited Cl-bearing phases, such as Cl-bearing hydrocalumite, AlOCl, and aluminum chloride hydrate, unlike D40. S45 produced C_4_AH_13_, unidentified phase, and Cl-bearing phases when compared with D45. For S50, the Cl-bearing phases and gismondine were formed, but they were not observed in D50. It is believed that the reaction products in seawater-mixed AAS samples was helpful in preventing the water percolation, and thus, the electrical resistivity increased compared with the deionized water-mixed sample. As the increase of electrical resistivity indicates the decrease of porosity in samples, it is expected that the seawater-mixed AAS pastes would exhibit better durability than the deionized water-mixed pastes.

## 4. Conclusions

This study investigated the characterization of early-age AAS pastes mixed with deionized water or seawater using electrical resistivity and by compared the properties of the hardened AAS pastes. For each AAS paste with seawater or deionized water, there was no significant change in the rising time with an increase in the *a/b* ratio, but the setting time was delayed and the compressive strength decreased. The delayed setting time is similar to the consequence of increasing electrical resistivity after rising time. The increasing ratio of electrical resistivity between *a/b* 0.45 and 0.5 was larger than that between *a/b* 0.4 and 0.45. This suggests that an optimized *a/b* ratio is between 0.4 and 0.45 for early age activation of GGBFS within 24 h.

The type of mixing water did not affect the setting time of AAS pastes. However, the increasing ratio of electrical resistivity and value of electrical resistivity were affected by the type of mixing water. For the same *a/b* ratio, the seawater-mixed AAS pastes produced higher electrical resistivity and early compressive strength than the deionized water-mixed pastes in AAS system. This was attributed to a higher degree of hydration in the seawater-mixed AAS paste.

The reaction products commonly identified in deionized water-mixed and seawater-mixed AAS paste were C–S–H(I), C–S–H, C–A–S–H, Ca(OH)_2_, K_2_SO_4_, hydrocalumite, and hydrotalcite. In comparison with the deionized water-mixed pastes, the increase in the electrical resistivity at approximately 24 h hydration time in seawater-mixed pastes may be attributed to the formation of Cl-bearing phases, such as Cl-hydrocalumite, AlOCl, and aluminum chloride hydrate. For all samples (mixed with deionized water and seawater), the electrical resistivity and early compressive strength of AAS pastes followed the same order: D50 (lowest) < D45 < S50 < D40 < S45 (highest), except for S40 and S45. The electrical resistivity of S45 was higher than that of S40, while the compressive strength of S45 was lower than that of S40. This suggests that although C_4_AH_13_ and some unidentified crystalline phase, which were newly formed in S45 compared with S40, are helpful in preventing the water percolation within the sample, they do not lead to the strength development.

## Figures and Tables

**Table 1:** Chemical composition (oxides in wt.%) of ground-granulated blast-furnace slag (GGBFS).

CaO	SiO_2_	Al_2_O_3_	K_2_O	SO_3_	Fe_2_O_3_	MgO	Na_2_O	TiO_2_	MnO	Others
43.02	32.20	13.59	0.61	4.77	0.51	3.68	0.28	0.70	0.46	0.19

Note: Others include SrO, BaO, and ZrO_2_.

**Table 2:** Chemical analysis of seawater (ppm).

Ca^2+^	K^+^	Mg^2+^	Na^+^	Cl^–^	SO_4_^2−^
380	380	1200	10,000	17,000	1900

**Table 3:** Mixture proportions of samples.

Sample Label	*a/b*	Activator (g)		Binder (g)
		4 M KOH in Deionized Water	4 M KOH in Seawater	GGBFS
D40	0.4	1000	-	2500
S40		-	1000	2500
D45	0.45	1125	-	2500
S45		-	1125	2500
D50	0.5	1250	-	2500
S50		-	1250	2500

**Table 4:** Rising time and increasing ratio of electrical resistivity in alkali-activated ground-granulated blast-furnace slag pastes.

Sample ID	Rising Time (h)	Increasing Ratio (Ωm/h)
D40	2.78	5.67
D45	2.95	5.43
D50	3.55	4.40
S40	3.10	6.24
S45	2.97	6.30
S50	3.32	5.51

**Table 5:** Phase change in the XRD pattern in Figure 6.

Sample ID	D40	D45	D50
Crystalline Phase			
Akermanite * (PDF #35−0592)	O	≈	≈
Gypsum * (PDF #21−0816)	O	↓	↓↓
Anhydrite * (PDF #37−1496)	X	X	O
Calcite * (PDF #47−1743)	O	≈	≈
C–S–H(I) (PDF #29−0331)	O	↓	↑(similar to D40)
C–S–H (PDF #33−0306)	O	↑	≈
C–A–S–H (PDF #46−1405)	O	↓	↑(similar to D40)
Ca(OH)_2_ (PDF #44−1481)	O	≈	↑
K_2_SO_4_ (PDF #01−0939)	O	↑	↑↑
Hydrocalumite (PDF #14−0083)	O	≈	↓
Hydrotalcite (ICSD collection #6296)	O	≈	↓
C_4_AH_13_ (PDF #11−0203)	X	X	O

Note. * crystalline phase contained in the original raw GGBFS (Figure 1); O: presence of phase; X: absence of phase; ≈, ↓, and ↑: no change, decrease, and increase compared with the sample on the left, respectively; ↓↓ (↑↑): further decreased (or increased) than the sample on the left. The numbers in parentheses are ICDD PDF−2 or ICSD data of the identified phases.

**Table 6:** Phase change in the XRD pattern in Figure 7.

Sample ID	S40	S45	S50
Crystalline Phase			
Akermanite * (PDF #35−0592)	O	≈	≈
Gypsum * (PDF #21−0816)	O	↓	↓↓
Anhydrite *	X	X	X
Calcite * (PDF #47−1743)	O	≈	≈
C–S–H(I) (PDF #29−0331)	O	↓	↓↓
C–S–H (PDF #33−0306)	O	↓	≈
C–A–S–H (PDF #46−1405)	O	≈	↓
Ca(OH)_2_ (PDF #44−1481)	O	↓	≈
K_2_SO_4_ (PDF #01−0939)	O	↑	↑↑
Hydrocalumite (PDF #14−0083)	O	↓	↓↓
Hydrotalcite (ICSD collection #6296)	O	↓	↓↓
Cl-bearing hydrocalumite (ICSD collection #088617)	O	↓	↓↓
AlOCl (PDF #74−1864)	O	↓	↓↓
Aluminum chloride hydrate (ICSD collection #026139)	O	↓	↓↓
C_4_AH_13_ (PDF #11−0203)	X	O	X
Gismondine (PDF #81−1858)	X	X	O

Note. * crystalline phase contained in the original raw GGBFS (Figure 1); O: presence of phase; X: absence of phase; ≈, ↑, and ↓: no change, increase, and decrease compared with the sample on the left, respectively; ↓↓ (↑↑): further decreased (or increased) than the sample on the left. The numbers in parentheses are ICDD PDF−2 or ICSD data of the identified phases.
